# Preparing for an Artificial Intelligence–Enabled Future: Patient Perspectives on Engagement and Health Care Professional Training for Adopting Artificial Intelligence Technologies in Health Care Settings

**DOI:** 10.2196/40973

**Published:** 2023-03-02

**Authors:** Tharshini Jeyakumar, Sarah Younus, Melody Zhang, Megan Clare, Rebecca Charow, Inaara Karsan, Azra Dhalla, Dalia Al-Mouaswas, Jillian Scandiffio, Justin Aling, Mohammad Salhia, Nadim Lalani, Scott Overholt, David Wiljer

**Affiliations:** 1 University Health Network Toronto, ON Canada; 2 Michener Institute of Education University Health Network Toronto, ON Canada; 3 Institute of Health Policy, Management and Evaluation, Dalla Lana School of Public Health University of Toronto Toronto, ON Canada; 4 Vector Institute Toronto, ON Canada; 5 Patient Partner Program University Health Network Toronto, ON Canada; 6 Faculty of Medicine University of Toronto Toronto, ON Canada; 7 Office of Education Centre for Addiction and Mental Health Toronto, ON Canada

**Keywords:** artificial intelligence, patient, education, attitude, health data, adoption, health equity, patient engagement

## Abstract

**Background:**

As new technologies emerge, there is a significant shift in the way care is delivered on a global scale. Artificial intelligence (AI) technologies have been rapidly and inexorably used to optimize patient outcomes, reduce health system costs, improve workflow efficiency, and enhance population health. Despite the widespread adoption of AI technologies, the literature on patient engagement and their perspectives on how AI will affect clinical care is scarce. Minimal patient engagement can limit the optimization of these novel technologies and contribute to suboptimal use in care settings.

**Objective:**

We aimed to explore patients’ views on what skills they believe health care professionals should have in preparation for this AI-enabled future and how we can better engage patients when adopting and deploying AI technologies in health care settings.

**Methods:**

Semistructured interviews were conducted from August 2020 to December 2021 with 12 individuals who were a patient in any Canadian health care setting. Interviews were conducted until thematic saturation occurred. A thematic analysis approach outlined by Braun and Clarke was used to inductively analyze the data and identify overarching themes.

**Results:**

Among the 12 patients interviewed, 8 (67%) were from urban settings and 4 (33%) were from rural settings. A majority of the participants were very comfortable with technology (n=6, 50%) and somewhat familiar with AI (n=7, 58%). In total, 3 themes emerged: cultivating patients’ trust, fostering patient engagement, and establishing data governance and validation of AI technologies.

**Conclusions:**

With the rapid surge of AI solutions, there is a critical need to understand patient values in advancing the quality of care and contributing to an equitable health system. Our study demonstrated that health care professionals play a synergetic role in the future of AI and digital technologies. Patient engagement is vital in addressing underlying health inequities and fostering an optimal care experience. Future research is warranted to understand and capture the diverse perspectives of patients with various racial, ethnic, and socioeconomic backgrounds.

## Introduction

### Background

Artificial intelligence (AI) technologies are being rapidly adopted and implemented in health care settings to augment clinical decisions and the delivery of patient-centered care [1]. The use of AI applications presents a paradigm shift in health care and serves as a positive enabler for achieving the quintuple aims of health care [2]. In particular, AI applications have the potential to further integrate health equity and patient activation to ameliorate siloed and biased care, as advocated by the National Academy of Medicine [2,3]. Fostering a patient-centered culture that considers health equity entails continued partnerships with patients and encourages them to be coleaders of change within the clinical ecosystem [2]. This shift must emerge from the leadership and organizational levels and should include both a commitment to and development of strategic priorities, which include patient and family engaged care [2]. For instance, the Canadian Institute for Advanced Research urges the need for a collaborative and integrative effort to establish an AI for Health strategy to accelerate the adoption and scaling of AI-enabled technologies to provide compassionate and safe care [4]. The Canadian Institute for Advanced Research highlights the importance of including patient perspectives in the development, implementation, and evaluation of AI initiatives [4]. A few studies have reported that a co-design approach engaging patients and the public during the development process could enhance the accuracy, equity, and transparency of AI models [5-7]. Patients are key beneficiaries in the adoption and implementation of AI technologies in clinical settings; thus, engaging patients allows for diversity in perspectives, and their values and needs are included [8,9].

### Importance of Fostering Patient Engagement

Patient engagement is defined as an individual’s active involvement in the care decision-making process and collaboration with key stakeholders to build an equitable and sustainable health system [10,11]. Understanding patient perceptions is an initial step in fostering patient engagement and ensuring the responsible and safe use of these novel technologies in clinical care settings [8]. A recent survey conducted by the Biron Health Group in Quebec indicated that many residents were in favor of using AI technologies to address health system issues and optimize clinical innovations [12]. The study showed that 63% agreed that AI could prevent adverse outcomes, while 40% believed that it could be used to augment clinicians’ expertise and lead to profound changes in care [12]. Many papers focused on patient perspectives of AI in various medical specialties, such as cardiology, dermatology, and radiology, and how they conceptualize AI technology in health care [13-21]. Although there are several studies focused on understanding patient perspectives in relation to specific AI technologies, patients need to be engaged at different stages of the AI implementation process [9,22-24]. The long-term sustainability of AI technologies in clinical environments vastly relies on patient acceptance, which is influenced by their knowledge and perception of opportunities as well as risks associated with using AI solutions [15].

Despite the positive views on the potential of clinical applications of AI and the promise of AI, there are many fears and misconceptions that remain. A few studies have shown that patients expressed concern regarding the use of personal health records for profit or being distorted by hackers, as this could have an impact on their employment or insurance coverage [15,25]. Balthazar et al [25] contended that even when patients have an in-depth understanding and thoughts on the appropriate use of their personal health information, they may not be able to understand the foundational concepts of machine learning models to make predictions or discern the difference between terms such as privacy and confidentiality. Another significant concern noted in the literature is the systemic bias that can potentially be embedded in AI models and that can stigmatize or marginalize certain populations [7,8,25]. Patients’ perspectives on AI may differ based on their socioeconomic status, ethnicity, and vulnerability [25]. Furthermore, patient engagement helps to cocreate the health care system, address the underlying social determinants of health [2,26], and ultimately democratize access to AI innovations [5]. Thus, minimizing the consequences and concerns of AI technologies is pivotal in facilitating trust and ensuring the successful adoption of these tools in clinical practice.

Establishing patient trust becomes increasingly difficult in a rapidly evolving digital space with complex and less-transparent AI technologies [8]. Studies have asserted that even though AI can empower patients, the lack of transparency and explanation of processes owing to the *black box* phenomenon could diminish patients’ trust if the model is not reflective of current evidence, is biased, or is erroneous [27-30]. Notwithstanding the high accuracy and advancements in AI technologies, patients value human judgment when making care decisions [31]. Empathy, compassion, and trust play a significant role in forming the basis for augmenting patient-centered care and ensuring the sustainability of AI innovations [27,32]. It is vital for care providers to actively engage patients when making care decisions and foster a therapeutic relationship [32]. Kerasidou [32] highlighted that patients preferred to interact with health care professionals (HCPs) who both have clinical expertise and provide empathetic and compassionate care. An interpersonal care model allows HCPs to better understand and address individual needs and to build patients’ trust [7,32]. In addition, the literature emphasizes the importance of public perception and literacy in fostering trust and removing any potential misconceptions regarding AI [28]. Esmaeilzadeh et al [27] advocated for patient education to ensure that patients are prepared to make informed decisions and communicate effectively with their care providers. The authors underlined the importance of patients being active partners during the adoption and integration of AI innovations in their care [27]. Thus, patient engagement helps diminish the gap between patients’ expectations of AI technologies and their experiences with care providers [33].

### Current Landscape

 Cutting-edge technologies such as AI are poised to transform the health care system, as we slowly shift to a new revolution in the next era [20]. This shift is facilitated through medical education; however, there are gaps in its implementation across all levels of medical education. This includes the lack of standardization, varying levels of AI literacy among faculty, and limited infrastructure for embedding AI concepts within existing curricula [34]. There is a need for medical education to go beyond medical informatics and machine learning, enabling HCPs to operationalize these novel tools at the point of care [34]. Despite the use of AI to accelerate innovations in patient care and the need for patient voices, there is limited literature on patient engagement and their perceptions of how AI will affect care delivery, thus ensuring AI technologies are aptly integrated within the clinical environment and cultivating patient trust [9]. To put the needs of patients first in creating a healthier world using AI, the objective of this study was to elucidate patients’ perceptions of what skills they believe HCPs should have in preparation for this AI-enabled future and how we can better engage patients when adopting and deploying AI technologies in health care.

## Methods

### Study Design

A qualitative study design was used to elicit participants’ perceptions of the adoption and implementation of AI within the health care ecosystem.

### Ethics Approval

This study was approved by the University Health Network Research Ethics Board (ID:20-6148.2).

### Study Participants

A maximum purposive sampling approach was used to ensure that the participants represented various comfort levels with AI technology and contexts in which they received care. It was also used to gain insights into the diverse perspectives that should be considered when adopting and deploying AI technologies in clinical settings. Purposive sampling enables researchers to identify and select participants based on their ability to yield relevant information about a particular phenomenon [35,36]. Participants were recruited from a national group of approximately 25 patients via email invitations sent on behalf of the research team by education committee members of Canada Health Infoway. Participants who consented to participate in the interviews were asked to inform individuals within their networks via a snowball sampling approach [37]. The snowball sampling method was used to recruit additional participants, who may add valuable perspectives to the study and enable an in-depth understanding of the phenomenon. Individuals were eligible to participate if they were patients at any Canadian medical center (acute or long-term) and were able to provide informed consent.

### Data Collection

Semistructured interviews were conducted with patients on the web via Microsoft Teams, in following COVID-19 pandemic social distancing measures. An instructional designer and research associates who have experience in qualitative research methods conducted the interviews. In addition, the interviewers have formal education in health informatics (TJ), public health (SY), educational technology (MC), and educational and counseling psychology (MZ). A semistructured interview guide consisting of 13 open-ended questions was used to guide discussions (Multimedia Appendix 1). The interviewers probed participants when necessary to further explore and understand salient ideas. The participants’ level of comfort in sharing their perceptions and experiences determined the length of the interview. The interviews lasted approximately 17 to 48 minutes. The interviews were conducted until the researchers felt that no new ideas emerged and data saturation was achieved. Participants were offered an honorarium of CAD $50 (US $37.32) in the form of e-gift cards. Verbal informed consent was obtained before conducting interviews. All interviews were digitally audio-recorded, professionally transcribed, and deidentified. The transcripts were reviewed for accuracy by a research associate.

### Data Analysis

Reflexivity is crucial in qualitative research, as it enables researchers to position themselves and reflect on the biases, values, and experiences that they bring [38,39]. Recognizing the researchers’ perspectives and positionality, research rigor was asserted by providing a reflexive stance in the research process, including different viewpoints from the team. Seven members of the core research team participated in the coding and analytic process, including 4 research associates from the digital education department at a large multisite academic health sciences center (TJ, SY, MZ, and SB), instructional designer (MC), 2 patient partners (JA and SO), and a senior investigator (DW, a PhD education researcher). This enabled a rigorous interpretation and analysis of the findings. A systematic process outlined by Braun and Clarke [40] was used to inductively analyze the data. Two research associates (TJ and SY) independently analyzed the first 3 transcripts from an exploratory lens and developed an initial coding structure. Each of the remaining transcripts were coded independently by two study team members (TJ and MC, MZ or SB). New data were constantly compared with the existing data, thus resulting in iterative refinement of the coding structure and the structuring of further data collection. Iterative discussions with the research team helped contextualize the overarching themes and resolve disagreements. The senior investigator (DW) on the team reviewed all themes and provided additional input when consensus could not be reached. Two patient partners who were part of the study team (JA and SO) reviewed the themes, which allowed for triangulation of the data from various perspectives. Data were analyzed for emerging themes using NVivo version 12 (QSR International), a qualitative data analysis software program. The rigor and quality of thematic analysis were evaluated using a 20-question evaluation tool [41]. The team also maintained a record of each team member’s coding, notes from meetings, and different versions of the coding structure. This review enhanced the credibility and trustworthiness of the findings. Furthermore, an intercoder agreement was established using NVivo 12 to ensure transparency and rigor of the data.

## Results

### Overview

In total, 12 interviews were conducted between August 2021 and December 2021. Of the 12 participants, 10 (83%) were females, and 2 (17%) were males. Table 1 shows the characteristics of the study participants. The average length of the interviews was 30 minutes. Most participants were very comfortable with the technology and somewhat familiar with AI. Thematic analysis of the data yielded three major themes, each with several subthemes (Table 2): (1) cultivating patients’ trust, (2) fostering patient engagement, and (3) establishing data governance and validation of AI technologies.

**Table 1:** Participant characteristics (N=12).

Characteristic				Value, n (%)
**Demographics**				
	**Age (years)**			
		Young adult (18-40)	0 (0)	
		Middle age (40-60)	7 (58)	
		Senior (≥60)	5 (42)	
	**Sex**			
		Male	2 (17)	
		Female	10 (83)	
	**Location**			
		Urban	8 (67)	
		Rural	4 (33)	
**Comfort with technology**				
	Not at all comfortable		3 (25)	
	Somewhat comfortable		3 (25)	
	Very comfortable		6 (50)	
**Familiarity with AI^a^**				
	Not at all familiar		3 (25)	
	Somewhat familiar		7 (58)	
	Very familiar		2 (17)	
**AI information source**				
	Family and friends		4 (33)	
	Career		5 (42)	
	Scholarly articles		2 (17)	
	Non–peer-reviewed articles		3 (25)	
	Social media		2 (17)	
	Other		3 (25)	
**Medical care**				
	**Frequency of visiting an HCP^b^**			
		Once a year	2 (17)	
		Fewer than 4 times a year	2 (17)	
		4 to 6 times a year	8 (66)	
	**Type of HCP**			
		Cardiologist	3 (25)	
		General practitioner	10 (83)	
		Ophthalmologist	3 (25)	
		Physiotherapist	3 (25)	
		Surgeon	3 (25)	
		Other	8 (67)	

^a^AI: artificial intelligence.

^b^HCP: health care professional.

**Table 2:** Summary of key themes.

Theme and quote				Significance
**Theme 1: cultivating patients’ trust**				
	**Subtheme: providing safe and compassionate care**			
		“I would feel comfortable as long as I still had a voice. And they listen to the voice, OK, as opposed to the data... I mean, if I trust my health care provider and they’re thorough and reliable, I would go along with it.” [ID 8] “I mean, I think I would worry about us totally removing the human part of this. That compassion and connection with a person who understands your health condition is really important... I would like a person who understands the question that I’m asking. So, I think it’s making sure that we don’t undervalue the importance of connection to other human beings, especially when we’re talking about health care and the fears and anxieties that come up, about our health, so that we have someone who can not only answer our questions, but understand our fears and worries...” [ID 9]	Transparent communication and acknowledging patient concerns and needs are imperative in fostering patients’ trust. Most importantly, participants seemed critical of the use of digital technologies and their impact on therapeutic relationships. Compassion was identified as crucial in achieving patient-centered care; and ensuring the presence of technology does not encumber the human and relational aspects of a patient-provider relationship.	
	**Subtheme: achieving transparency in care decisions**			
		“I want to know for sure like that it’s a legitimate app that it’s recommended by like major hospitals and those sorts of things, because right now everybody’s making apps and it’s very hard to tell what’s real and what’s not, especially at my age. I find my generation, my husband, we’re much less trusting and we get confused, like the example of that bot that I was very unhappy with the bot being there [instead of a person]. But I would also be good if, let’s say there are apps that it was overseeing. So, with a hospital, those sorts of things, like I really would like proof. And if it was dealing with my physician, well, then having her backing that would make me feel more comfortable using the app as well.” [ID 2]	Given the rapid proliferation of digital technologies for patient care, participants stressed the need to be governed or regulated by the organization for the privacy and legitimacy of the app.	
**Theme 2: fostering patient engagement**				
	**Subtheme: enabling patients to be coleaders in their care**			
		“The only thing I would say at the outset would be it’s the machine that is running the process and I would want to be assured that the patient’s feelings and voice would still be heard. Because there are things that, you know, there are things maybe ninety-nine percent going one way, but there is still that one percent that maybe the patient feels. Maybe there’s other things going on with that patient that would come out in a meeting with a doctor.” [ID 8] “I think it’s important that we as patients are as involved in our care as possible. I would like to expect that my GP would engage me in the decision-making about my care, even if an algorithm directed him to do something or not do something, I think that’s an important aspect of communication.” [ID 4]	Participants highlighted the importance of HCPs^a^ engaging them during the clinical decision-making process and providing an opportunity for them to share their thoughts and perspectives.	
	**Subtheme: increasing confidence among patients**			
		“Some people will want to know a lot and some people will want to know less. But certainly, the overall importance of sharing on some level so that we can improve our systems, I think is critical, but how do we do it safely? And if we can explain that to people in a way that gives them confidence and that they know their information will not be released to the wrong people in an identifying way, that’s important, but it doesn’t obviate the risk completely. So, I still think that you know, people need to at least have the opportunity to understand that this is a really complicated and important decision to make… how could that information be used in ways that are contrary to your best in financial health or otherwise?” [ID 4] “…a health professional who can also help me and guide me if there’s something that I don’t understand, or I’m missing a piece of this puzzle. So, a coach and educator. Yeah, someone who’s got my back with the AI as well. So again, I just think we can’t lose sight of that human touch and how we learn and digest and understand information. It’s not just a transaction.” [ID 9]	When using technologies at the point of care, HCPs need to explain to patients the benefits and risks associated with it; thus, enabling them to be informed and understand how decisions are made. One participant emphasized that the patient-provider interaction is not a transaction as the technology can become a third player and the provider may neglect the compassionate aspect of the relationship. In addition, educating patients on the fundamentals of AI^b^ and other technologies can increase their confidence.	
**Theme 3: establishing data governance and validation of AI technologies**				
	**Subtheme: responsibility of data stewards**			
		“…I have strong objections to it being sold. I know the [organization] was making their data available to a private company at one point. And I know there are doctors in Ontario who feel that the health record is theirs, and they own it. And so, the information and it may be mine, but since they own the program that holds my data, they feel they have every right to sell it, and they do. So, I want more control over who gets to use it and why. And I mean, I think a lot of people would say, I’m fine for the public good, I’m fine with research that will benefit me, and people like me. But they draw the line at people making money from their personal data.” [ID 10] “I would like to know if there’s any third parties going to see it. My other concern… Say the insurance, I tested positive for breast cancer, and it was a genetic one, I’m going through that right now. What how having AI and data out there on a computer without being shared with insurance companies, which is more likely to happen than it is right now. So, yeah, I would want to know how my privacy is being respected. And any third parties involved and any changes I’d want to be updated and if there were changes and third parties were going to see it, I’d have the choice of letting them or completely removing all my information.” [ID 2]	Participants expressed concerns regarding how their data will be used and who will have access to it. They identified the need to provide them with a choice to opt-in or opt-out of the secondary use of data.	
	**Subtheme: quality assurance and validation of AI technologies**			
		“Then that becomes no different if there’s no oversight or no background or no warnings about them or disclaimers, then it becomes just the same as people Googling everything. So, I would want it to be a better tool and a somewhat regulated tool or something so that it’s actually endorsed by the medical community before it’s available, or at least obviously they’re not going to be able to control everything that’s available on the Internet. But at least there would be some education to the public that to use the tools that we endorse or use the tool endorsed by your hospital or your province or whatever, there would be some kind of oversight. That’s all I’m concerned about, that it just becomes the next version of Google.” [ID 7]	Quality assurance and validation of AI technologies are pivotal in ensuring the confidentiality of patient data and protecting them against nefarious acts.	
	**Subtheme: ensuring AI technologies used in clinical contexts are equitable and inclusive**			
		“Oh, yeah, definitely as a tool to assist physicians, I think it would be great. And I think that there are circumstances where the artificial intelligence tool might do a better job than the doctor. Because you know, a lot of people in health care are… people have preconceived notions about them, right. For instance, if somebody decides that you’re a hysterical woman, you won’t get the same care as you would if you had didn’t have that notation in your health record. And so, I think that with the use of artificial intelligence, it takes out some of the bias.” [ID 10] “I guess it really depends on who has actually set up the AI and what biases they have and what has actually been programmed into the system and if that’s actually missing data, just because of the bias and missing marginalized populations or people that don’t have a lot of money or are of a different race. And look, I just think there was something that I saw a while back about an app, you know, telling somebody had heart attack symptoms, and if it was male, it would say you should go to the hospital. But if it was female, it was like, oh, you don’t have a heart attack. You have I’m guessing this was a while ago, I’m guessing probably anxiety! So, there’s like [sex] differences, too. And so, I just wonder about the disparities that could be created, if it hasn’t been created with the people that it’s looking at.” [ID 5]	One of the participants stated that AI could be an unbiased tool for HCPs to use in their care as it removes some of the preconceived perceptions that lead to further marginalization of certain groups. HCPs need to become adept in examining and acknowledging implicit biases to make informed decisions and prevent unintended consequences on patient care.	

^a^HCP: health care professional.

^b^AI: artificial intelligence.

### Theme 1: Cultivating Patients’ Trust

#### Providing Safe and Compassionate Care

Most participants believed that trust is fundamental to ensuring that AI technologies are successfully integrated into clinical care settings. They would be comfortable using an AI-based application if they knew it was coming from a trusted source such as their health care provider. However, they also mentioned that they would feel uncomfortable if they did not have the opportunity to discuss the technology with their health care provider or did not have a follow-up conversation with them:

I would feel comfortable as long as I still had a voice. And they listen to the voice, OK, as opposed to the data...I mean, if I trust my health care provider and they’re thorough and reliable, I would go along with it.

ID 8

Using this technology in conjunction with the clinician’s expertise helps foster trust and ensures greater accountability. A few participants asserted that they would prefer their care provider to use their own knowledge and experience to make an informed decision and not solely based on the technology itself. As technologies are being integrated into clinical settings, patients do not want anything to change in the way they interact with their care provider or the way in which information is provided:

I mean, I think I would worry about us totally removing the human part of this. That compassion and connection with a person who understands your health condition is really important...I would like a person who understands the question that I’m asking. So, I think it’s making sure that we don’t undervalue the importance of connection to other human beings, especially when we’re talking about health care and the fears and anxieties that come up, about our health, so that we have someone who can not only answer our questions, but understand our fears and worries.

ID 9

Participants indicated that face-to-face interactions and the clinician’s presence are important for creating a safe space and maintaining trust. Participants commented that having a conversation with a clinician, as opposed to only interacting with the AI technology, provides support and reassurance, particularly when discussing sensitive health concerns such as mental health issues.

#### Achieving Transparency in Care Decisions

Participants would like clear communication from their HCPs on what applications and analytic health care tools are available and whether they are being used in their care. The participants expressed their desire for transparency in how physicians combined their judgment and technology to arrive at diagnoses and care decisions. Other participants noted that care providers did not have to understand the technical aspects of AI technology but needed to be confident in what they are prescribing and practicing to ensure that it is safe for patients.

Several participants also reported that care providers who willingly answered their questions or demonstrated ways to interact with the technology significantly increased their confidence levels in the technology. One participant mentioned that in comparison with providers who chose not to explain or demonstrate an AI technology, having an HCP explain what they did greatly boosted a patient’s positive perception of the technology and their comfort with it. Some participants also preferred to see how physicians interacted with the technology and process they used to make clinical decisions. Furthermore, patients would prefer guidance on using health technologies and ascertaining what information is relevant to their own health care. One participant mentioned that they liked information on how the backend technology of an AI-enabled mobile application (app) was created. Regardless of the degree to which patients wanted to understand how an app works, they conveyed the need for any apps used to be vetted and recommended by their HCP:

I want to know for sure like that it’s a legitimate app that it’s recommended by like major hospitals and those sorts of things, because right now everybody’s making apps and it’s very hard to tell what’s real and what’s not, especially at my age. I find my generation, my husband, we’re much less trusting and we get confused, like the example of that bot that I was very unhappy with the bot being there [instead of a person]. But I would also be good if, let’s say there are apps that it was overseeing. So, with a hospital, those sorts of things, like I really would like proof. And if it was dealing with my physician, well, then having her backing that would make me feel more comfortable using the app as well.

ID 2

Differences were found in the level of knowledge patients want to know about how AI technologies or apps work and the potential impacts on care decisions. However, all participants expressed the importance of transparency and communication in an app or provider’s process for making care recommendations or decisions. Patients also want to be informed of the AI technologies that exist and whether they should be used in their care. Although there was a difference in the level of knowledge patients wanted their HCPs to have, the participants emphasized comfort in their recommendations and transparency.

### Theme 2: Fostering Patient Engagement

#### Enabling Patients to Be Coleaders in Their Care

Enabling patients to become coleaders is vital when using digital technologies to inform care decisions. Participants asserted that it is important for health care organizations to actively listen to and understand the needs of the public:

The only thing I would say at the outset would be it’s the machine that is running the process and I would want to be assured that the patient’s feelings and voice would still be heard. Because there are things that, you know, there are things maybe ninety-nine percent going one way, but there is still that one percent that maybe the patient feels. Maybe there’s other things going on with that patient that would come out in a meeting with a doctor.

ID 8

Two participants specifically mentioned that they would like to be engaged and involved in the shared decision-making process, which also helps foster trust. For instance, if the AI application detects a concern, the patient would expect the care provider to have a discussion with them to identify the next steps:

I think it’s important that we as patients are as involved in our care as possible. I would like to expect that my GP would engage me in the decision-making about my care, even if an algorithm directed him to do something or not do something, I think that’s an important aspect of communication.

ID 4

Participants reported that the integration of digital solutions as part of patient care is contingent upon the relationships they have established with their HCPs.

#### Increasing Confidence Among Patients

In the use of an AI app or technology, participants expressed the need for a log-in ID; a password; and an accessible, easy-to-use interface. They commented that having access to technology, such as being able to view the results on a cloud platform or digital patient profile, would be valuable and aid in their decision-making process. Furthermore, participants highlighted the need for patient education:

Some people will want to know a lot and some people will want to know less. But certainly, the overall importance of sharing on some level so that we can improve our systems, I think is critical, but how do we do it safely? And if we can explain that to people in a way that gives them confidence and that they know their information will not be released to the wrong people in an identifying way, that’s important, but it doesn’t obviate the risk completely. So, I still think that you know, people need to at least have the opportunity to understand that this is a really complicated and important decision to make...how could that information be used in ways that are contrary to your best in financial health or otherwise?

ID 4

Although patients do not need to understand all details of their diagnosis, it is essential to provide them with relevant information at the right level. Participants reported that education helps increase awareness of existing AI technologies and how these technologies are used to augment patient care. Another participant stated that it would be beneficial if medical professionals provided support and allocated some time to help patients understand the AI technologies being used in clinical practice. Hence, understanding the fundamentals underpinning AI technology helps foster confidence among patients and increases their appreciation for the support provided by the technology:

A health professional who can also help me and guide me if there’s something that I don’t understand, or I’m missing a piece of this puzzle. So, a coach and educator. Yeah, someone who’s got my back with the AI as well. So again, I just think we can’t lose sight of that human touch and how we learn and digest and understand information. It’s not just a transaction.

ID 9

An intuitive, interactive AI app or technology was also mentioned as an important element of confidence. When patients use technology as part of their care, they want to ensure that their concerns, thoughts, and opinions are heard. When their care provider was not physically present, patients expressed the desire for a connection. That is, despite the lack of a physical presence, patients preferred using a technology with interactive features to respond to their questions or concerns.

### Theme 3: Establishing Data Governance and Validation of AI Technologies

#### Responsibility of Data Stewards

Participants expressed privacy concerns, such as how their health data would be used and shared, and for what purposes. In particular, participants mentioned fear of their personal data in apps being sold to private companies or used for illicit purposes:

I have strong objections to it being sold. I know the [organization] was making their data available to a private company at one point. And I know there are doctors in Ontario who feel that the health record is theirs, and they own it. And so, the information and it may be mine, but since they own the program that holds my data, they feel they have every right to sell it, and they do. So, I want more control over who gets to use it and why. And I mean, I think a lot of people would say, I’m fine for the public good, I’m fine with research that will benefit me, and people like me. But they draw the line at people making money from their personal data.

ID 10

Participants voiced several concerns about the privacy of their health data and its potential for long-term use when entering web-based portals or apps. Many participants suggested the importance of choice regarding the types of information used for secondary purposes. They also expressed value in having the option to accept or reject the use of their information by third parties and to be able to remove their data, if desired. One participant worried about long-term consequences, such as familial genetic records being attached to future generations and potential lifetime implications from youth sharing personal information on mental health chatbots. Another felt it was important to understand how their health data were used to augment AI and its financial implications. Patients also wanted to be informed of how their health data would be protected, how to access their own data, who had access to it, and potential long-term consequences. Gatekeepers were identified as critical in ensuring the compliance and security of patient data as well as managing any regulatory risks:

I would like to know if there’s any third parties going to see it. My other concern...Say the insurance, I tested positive for breast cancer, and it was a genetic one, I’m going through that right now. What how having AI and data out there on a computer without being shared with insurance companies, which is more likely to happen than it is right now. So, yeah, I would want to know how my privacy is being respected. And any third parties involved and any changes I’d want to be updated and if there were changes and third parties were going to see it, I’d have the choice of letting them or completely removing all my information.

ID 2

Informed consent to access data, disclosure of use, and potential risks were stated as critical measures to protect patient privacy. Data protection and security were emphasized as key mitigation steps to ensure that patient data would not be disclosed. If data were shared without consent or accidentally, participants expressed the need for legal barriers, so that third-party companies would have no recourse. Participants desired apps to be verified by trusted sources, such as hospitals and the government, with transparency on the backend technologies deployed within them and how their data would be handled.

#### Quality Assurance and Validation of AI Technologies

Interestingly, participants also highlighted the need to understand more about how health care systems benefit from investment in AI technologies. They reported that this would help deliver care more effectively through the use of preventive tools and by identifying optimal treatment options. Some participants argued that AI technologies could contribute to additional health expenditures and further amplify the pressure on an overburdened health care system. In a public health system, it is essential to maximize benefits across the system and reduce costs.

Moreover, participants reported the need for governance and oversight in terms of quality of assurance and accessibility of technology. Participants emphasized that there should be a governing body that evaluates the technologies used in clinical care before endorsing them:

Then that becomes no different if there’s no oversight or no background or no warnings about them or disclaimers, then it becomes just the same as people Googling everything. So, I would want it to be a better tool and a somewhat regulated tool or something so that it’s actually endorsed by the medical community before it’s available, or at least obviously they’re not going to be able to control everything that’s available on the Internet. But at least there would be some education to the public that to use the tools that we endorse or use the tool endorsed by your hospital or your province or whatever, there would be some kind of oversight. That’s all I’m concerned about, that it just becomes the next version of Google.

ID7

Participants preferred a regulated technology that was validated by the medical community before being available to the public. One participant mentioned that, without regulation, random apps would be produced and sold to hospitals.

#### Ensuring AI Technologies Used in Clinical Contexts Are Equitable and Inclusive

Participants would like to understand how AI technologies will be used in their health care, who would be using them, and for what reasons. One of the participants also mentioned that AI could be an unbiased solution for physicians to use in their care:

Oh, yeah, definitely as a tool to assist physicians, I think it would be great. And I think that there are circumstances where the artificial intelligence tool might do a better job than the doctor. Because you know, a lot of people in health care are...people have preconceived notions about them, right. For instance, if somebody decides that you’re a hysterical woman, you won’t get the same care as you would if you had didn’t have that notation in your health record. And so, I think that with the use of artificial intelligence, it takes out some of the bias.

ID 10

Some participants reported the use of biased data for model development and the lack of diversity represented in data sets as problematic. Inherent biases are sometimes created when data sets are not heterogeneous, which can exclude vulnerable populations. Sex and racial disparities, for instance, can also be created if inherently biased data are included in data sets and applications:

I guess it really depends on who has actually set up the AI and what biases they have and what has actually been programmed into the system and if that’s actually missing data, just because of the bias and missing marginalized populations or people that don’t have a lot of money or are of a different race. And look, I just think there was something that I saw a while back about an app, you know, telling somebody had heart attack symptoms, and if it was male, it would say you should go to the hospital. But if it was female, it was like, oh, you don’t have a heart attack. You have I’m guessing this was a while ago, I’m guessing probably anxiety! So, there’s like sex differences, too. And so, I just wonder about the disparities that could be created, if it hasn’t been created with the people that it’s looking at.

ID 5

The participants stressed the importance of ensuring that the training and testing data sets are heterogeneous and representative of the target population. Acknowledging these biases enables clinicians to make informed decisions and prevent any unintended consequences of patient care.

## Discussion

### Principal Findings

As new technologies and AI solutions emerge within health care, it is crucial to ensure that patients are included in the delivery of their own care. Advancements in digital technologies have revolutionized the possibilities of delivering optimal and patient-centric care in this continuously evolving health care ecosystem. Despite the rapid penetration of innovative technologies in clinical care, little is known about the effectiveness of AI technologies. The efficacy and long-term adoption of these technologies depend greatly on patient engagement and adherence [15]. McMahon [42] contended that patient engagement as part of medical education and continuing professional development is crucial in providing an opportunity for HCPs to develop their patient-centric skills, increase sensitivity to patient needs and values, and foster interprofessional collaborative practice. Patient expertise is based on their unique experiences of receiving care and the impact of the social determinants of health. Therefore, it is important to acknowledge and appreciate the value of these diverse patient viewpoints [43,44]. In addition, patient participation is reported to improve care providers’ communication skills and empathy and increase their awareness of patients’ needs in marginalized communities [45-47].

This study aimed to understand patients’ perspectives on how to better foster patient engagement in the uptake of AI technologies and what competencies they believe are essential in preparing HCPs for digital care. Through semistructured interviews with patient partners, three predominant themes emerged: (1) cultivating patients’ trust, (2) fostering patient engagement, and (3) establishing data governance and validation of AI technologies. Participants in both urban and rural settings highlighted similar ideas with regard to AI adoption.

In a recent scoping review, Charow et al [34] identified key competencies that are currently taught as part of the AI curriculum and what programs should be taught. The authors used Bloom Learning Taxonomy to group curriculum topics [34]. Table 3 illustrates the overlap of competencies identified in the scoping review and highlighted by the participants in this study.

As technologies are being integrated within care settings, participants in this study emphasized that it is important for HCPs to acknowledge how data are acquired and processed and explain a rationale when making decisions. Interestingly, the psychomotor and affective domains of Bloom Learning Taxonomy were reiterated by participants. Critical appraisal, ethical and legal considerations, communication, interpersonal skills, empathy, compassion, and emotional responsiveness were highlighted as important competencies to minimize the negative implications of AI integration at the point of care.

This study highlights the importance of establishing trust and transparency as part of the patient-clinician relationship. Many participants stated that lack of transparency in data access and use could potentially erode their trust in using AI for care delivery. This was in line with a previous study [30], which suggested that physicians must have a thorough knowledge of the AI technologies used and be prepared to provide a coherent rationale when making clinical decisions. For instance, if a patient is diagnosed with cancer, they would want to understand how AI technology arrived at that decision [48]. What becomes a challenge, however, is that advanced AI technologies are often built using complex algorithms, which may be difficult to explain, even if clinicians have the technical expertise [48]. In a qualitative study that examined patient privacy perspectives on health information exchange, trust was identified as a key antecedent for establishing effective patient-clinician relationships [49]. Transparent communication regarding the use of AI technologies serves as an initial step toward cultivating trust [49]. The authors noted a significant association between patients’ trust in clinicians and their willingness to share personal health information [49].

Patients believe the clinician’s presence is important, particularly when discussing sensitive information regarding their care. AI technologies should support existing patient care and not replace physician interactions. Similar to our study, previous research indicated that patients valued the interaction with the clinician rather than with AI technology alone [29]. AI technologies can potentially diminish clinician-patient interactions and jeopardize the humanistic facet of patient care [15,50]. Patients who interacted only with AI technologies in their care reported a lack of compassion and empathy [19,21,22] and a limited opportunity for patients to ask follow-up questions, discuss treatment options, and receive emotional support [19,21]. Davenport and Kalakota [48] further reinforced this point, highlighting the importance of establishing an empathetic relationship between clinicians and patients. In other studies, patients specified that the AI output should be verified by the physician for accuracy [22] and be used as a second opinion to inform clinical decisions [9,19]. In the event of a disagreement between the physician and the AI technology, patients favor the physician’s judgment as the final decision [9,22]. Yang et al [21] reported that AI can serve as a copilot in automating tasks and optimizing the quality of care. More importantly, the literature emphasizes the role of providers in decision-making, as they need to adapt the AI results based on the uniqueness of each patient and their circumstances [9].

Engaging patients in proactive care leads to better patient experience and improved health system outcomes [48]. The findings from this study suggest that education on AI innovations helps to create awareness and foster confidence among patients. As a result, patients’ self-efficacy increases, enabling them to be knowledgeable and competent in safely navigating a digitized health care environment. This also contributes to the increased acceptance of AI technologies in practical settings to enhance the quality of care. Recent studies on patient perspectives on the use of AI in health care reported that it is critical for patients to be educated on the threats of AI technologies in an ever-increasing technology-enabled care environment [50,51]. Cultivating a strong culture of cybervigilance across this new digital space is vital for delivering care and ensuring that large amounts of sensitive and valuable data in vulnerable systems are protected. Moreover, Kovarik [52] reported that patients should be educated on the fundamentals of AI, which will be valuable when discussing diagnoses and treatment options.

Furthermore, the findings of this study underline the need for data stewards and regulations to ensure the protection and confidentiality of patient data. Consistent with previous literature, patients reported high levels of concern toward the misuse of their personal health information [15,48,51]. Patients in this study also expressed privacy concerns, such as how their health data would be used, how their data would be shared, and for what purposes. This ambivalence has resulted in increased fear among patients, and the need for choice and autonomy. Participants stated that it was important to have a choice in terms of consenting to what information they would prefer to opt-in or opt-out for secondary use of data. In a review article on the practical implementation of AI technologies, the authors asserted that cybersecurity measures need to be implemented to address concerns about the inappropriate use of patient data [53]. A few studies have reported that patients feared that their personal health information might be not anonymized or be used for profit by insurance and third-party companies [15,50]. In one study, patients perceived that insurance companies could use AI technologies to discern new information about their health and make changes to their premiums [9].

Oversight and regulatory measures are necessary to ensure the confidentiality of patient data and to protect against nefarious acts [9]. The AI implementation toolkit developed by Canada Health Infoway provides guidance on an AI governance framework [54]. This framework consists of 3 key constructs that oversee the responsible and ethical implementation of AI technologies: people, policies, and procedures [54]. The people construct consists of skillsets required to form a committee that provides procedural and practical guidance for AI implementation [54]. Policies focus on providing directions for risk considerations related to AI [54]. Procedures provide operational guidance on implementation aspects, including risk assessment, data testing, and monitoring [54]. Establishing governance structures is pivotal in monitoring ethical issues and mitigating any negative repercussions as a result of AI implementation in a milieu of increasing vulnerability to data breaches [48]. Matheny et al [3] delineated that it is imperative to involve patients and their families when developing regulatory and legislative solutions regarding the use of AI technologies in clinical contexts.

Finally, the participants noted the importance of examining implicit biases to ensure that AI technologies are inclusive and equitable. Biases in data sets may pose challenges in generalizing results and further exacerbate health inequities as well as discriminatory practices. This point was reinforced in a nominal group technique study that emphasized the negative implications of using homogenous data sets for developing algorithms [23]. One example of this is when AI models are developed based on data from a single health care institution, which may not be representative of a larger population [55]. The literature also reports that developers could inadvertently integrate their biases into the model development process [9]. Daneshjou et al [56] noted that there are no standards for describing data sets used for AI model development. Descriptions of data sets could aid in a better understanding of models and any underlying biases. Interestingly, our study also accentuated the notion of using AI technologies to reduce bias from a patient perspective. In health care, clinicians sometimes have preconceived notions about their patients; hence, a patient may not receive the same care as they would if they did not have that notation in their health records. Participants believed that AI technologies could remove some of the preconceived ideas and perceptions that contribute to the marginalization of specific populations when providing care, thus creating a more equitable and inclusive care environment.

**Table 3:** Overlap of competencies identified in the scoping review and highlighted by participants in this study.

Bloom taxonomy domain	Competencies identified in the scoping review (Charow et al [34])	Competencies highlighted by participants in this study and the scoping review (Charow et al [34])
Cognitive	Fundamentals of AI^a^ Implementation of AI Big data Data science, machine learning, and statistics Multidisciplinary collaboration Strengths and limitations of AI Predictive analytics Economic considerations EHR^b^ fundamentals	Ethics and legal issues Data governance
Psychomotor	Analytical Problem solving Product development Data visualization	Interpretation Communication Critical appraisal Medical decision-making Cultivation of compassion and empathy
Affective	Change management Adoption of AI	Perceptions of humanistic AI-enabled care Create and sustain a culture of trust and transparency with stakeholders and patients

^a^AI: artificial intelligence.

^b^EHR: electronic health record.

### Limitations

The findings of this study should be examined in light of these limitations. A limitation of this study is that the study population included no individuals in the age range of 0 to 40 years. Despite the less frequent use of health care services in this age group, they may represent a more technology-savvy population. This study provides diverse perspectives from rural and urban settings in Canada, as context plays a pivotal role in influencing the uptake of technology. This study provides a nuanced understanding of patient perceptions in both settings and how their perceptions may be similar. The interviews were conducted until theoretical saturation was achieved (n=12). In addition, a rigorous analytical approach was adopted, including iterative discussions with the research team and patient partners to validate emerging themes. Another limitation of this study was the recruitment of predominantly female patients, contributing to an underrepresentation of male voices. Demographic data such as race, ethnicity, employment, disability, and language were not collected, as the purposive sampling attempted to recruit participants based on comfort with the technology and the contexts in which they received care.

### Conclusions

This study revealed that to successfully adopt AI technologies in care settings, it is crucial to foster patient trust, build continued partnerships with patients, and establish data governance and validation of AI technologies. As we shift to a digital form of care, AI innovations are being rapidly adopted and implemented within the clinical ecosystem at a fast pace to advance the delivery of patient care and enhance efficiency at a systems level. Rather than AI becoming a replacement for humanistic care, AI and care providers play a synergetic role in the future of digital care. Understanding the needs and values of patients helps ensure the safe, effective, and responsible use of AI. Patient engagement helps to provide a real-world perspective and coconstruct knowledge from an end-user standpoint, thus ensuring that AI innovations are successfully integrated into practice settings. The findings of this study have implications for all stakeholders with accountability to ensure that patients are actively engaged in sustaining safe and high-quality care.

## Appendix

Multimedia Appendix 1 Patient context.

## Abbreviations

AI: artificial intelligence
HCP: health care professional
