# Supplementary material for: Preparing for an Artificial Intelligence–Enabled Future: Patient Perspectives on Engagement and Health Care Professional Training for Adopting Artificial Intelligence Technologies in Health Care Settings
Source: JMIR AI. 2023 Mar 2;2:e40973. doi: 10.2196/40973 (PMC11041489; doi:10.2196/40973)
Supplement: Multimedia Appendix 1 [file ai_v2i1e40973_app1.docx]

**MULTIMEDIA APPENDIX 1:**

**Patient Context:**

1. How would you describe the stage of life you are in, e.g., young-adult, middle-aged, or a senior?
2. Would you say you live in an urban city or rural town?
3. How often do you typically receive medical care?
4. If you are comfortable, could you tell us what types of health care providers you see most often?
5. Prompt: such as a family doctor, psychologist, cardiologist, oncologist, nutritionist, physiotherapist
6. Can you describe how comfortable you are with technology and how you use technology to manage your health or health care?

**Perception of Artificial Intelligence (AI):**

Scenario: Artificial intelligence (AI) is a field in computer science where computers are programed and trained to think like humans. Through the training and testing process, the AI technology can learn and self-adapt based on new data and feedback. Computers that use AI can go through large amounts of data to find patterns and make predictions and decisions.

1. What do you think of when you think of Artificial Intelligence (AI)?
   1. Prompt: what words or feelings come to mind?
2. How much do you feel you know about AI?
   1. Prompt: how familiar are you with AI?
   2. Prompt: how familiar are you with computer science?
   3. Prompt: how familiar are you with statistics?
   4. Prompt: what type of sources do you hear about AI from?

Scenario: AI technologies could support your health care providers by giving them recommendations for different decisions they make about your care. These decisions could include scheduling, diagnosis support, and patient outcomes and preventative care.

For example, a team at Sunnybrook is working on an AI technology to help reduce the number of unnecessary biopsies for breast cancer. When the results of a scan are clearly negative or benign, no further test would be needed. Similarly, if it shows a high risk of cancer, a biopsy would be ordered. When the scan shows a low to medium risk of cancer, the decision about whether it needs to be biopsied is more subjective. The AI technology the team is building is being trained on sets of past MRI images, biopsy decisions, and biopsy results to help it recommend whether a patient should be sent for a biopsy. The AI recommendation could be included with the MRI results to help doctors make decisions about next steps.

This is one example of how AI technologies could be involved in your care. In addition to support for other diagnosis decisions, AI could help with scheduling and patient outcomes and preventative care decisions as well. The following questions will ask how you feel about technologies like these and what you would want to know. Do you have any questions about AI technologies your providers could use before we begin?

1. How comfortable would you feel if your health care providers used AI technologies like this to support your care?
   1. Prompt: what makes you feel comfortable?
   2. Prompt: what makes you feel uncomfortable?
   3. Prompt: what could make you feel more comfortable?
   4. Prompt: what level of knowledge would you want your health care providers to have about AI?
2. How would you like your health care provider to interact with you if they wanted to use an AI technology?
   1. Prompt: would you want your health care provider to tell you if they wanted to use an AI technology in your care?
   2. Prompt: when would you want your health care provider to tell you about how AI will be involved in your care?
   3. Prompt: how would you want your care provider to tell you the role AI will play in your care?
   4. Prompt: how would you want AI results and decisions communicated to you?
   5. Prompt: thinking of experiences with other technologies in your care, what might help you feel empowered in your care when AI technologies are used?
   6. Prompt: what aspects of your current care would you want to remain unchanged?
   7. Prompt: what aspects of your current care would you want to be changed?
3. What information would you want to know about an AI technology that your health care provider might use for your care?
   1. Prompt: what information would you want to know about how the AI technology was created?
   2. Prompt: what information would you want to know about how the AI technology works?
   3. Prompt: what information would you want to know about how the AI technology would be used?
   4. Prompt: what information would you want to know about how your health data would be used?
   5. Prompt: is there anything else you would want to know?

Scenario: AI-based apps could also support you in self-managing your health or chronic illnesses. It might use information you enter or link to the app and from approved medical databases. Some examples of these types of apps are symptom checkers, AI nurses, nutrition plans, and mental health chatbots.

For example, symptom checker apps could use AI to help you decide what steps to take based on symptoms or images you input into them. They compare your symptoms or images to databases of medical information to return the best-matched diagnosis. They might also recommend next steps to take and recommend best-fit health care providers.

This is one example of how AI-based apps could help you monitor your health or manage your care. The following questions will ask how you feel about technologies like these and what you would want to know. Do you have any questions about AI-based apps before we begin?

1. How comfortable would you feel using AI technologies like this to support your health care?
   1. Prompt: what makes you feel comfortable?
   2. Prompt: what makes you feel uncomfortable?
2. What information would you want to know about an AI technology that you could use to manage your health?
   1. Prompt: what information would you want to know about how the AI technology was created?
   2. Prompt: what information would you want to know about how the AI technology works?
   3. Prompt: what information would you want to know about how the AI technology could be used?
   4. Prompt: what information would you want to know about how your health data would be used?
   5. Prompt: is there anything else you would want to know?
3. How would you like to learn about these technologies?
   1. Prompt: what type of involvement would you want your health care providers to have?
   2. Prompt: how would you want your health care providers to recommend AI technologies?
   3. Prompt: what could your health care providers do to help you feel empowered in choosing technologies for your self-care?
